# Advanced or Metastatic Cutaneous Squamous Cell Carcinoma: The Current and Future Role of Radiation Therapy in the Era of Immunotherapy

**DOI:** 10.3390/cancers14081871

**Published:** 2022-04-07

**Authors:** Gianluca Ferini, Paolo Palmisciano, Stefano Forte, Anna Viola, Emanuele Martorana, Silvana Parisi, Vito Valenti, Corrado Fichera, Giuseppe Emmanuele Umana, Stefano Pergolizzi

**Affiliations:** 1 Department of Radiation Oncology, REM Radioterapia srl, Via Penninazzo 11, 95029 Viagrande, Italy; vito.valenti@grupposamed.com; 2 Department of Neurosurgery, UC Health, Cincinnati, OH 45209, USA; paolo.palmisciano94@gmail.com; 3 IOM Ricerca srl, Via Penninazzo 11, 95029 Viagrande, Italy; stefano.forte@grupposamed.com (S.F.); emanuele.martorana@grupposamed.com (E.M.); 4 Fondazione Istituto Oncologico del Mediterraneo, 95029 Viagrande, Italy; anna.viola@fondazioneiom.it; 5 Radiation Oncology Unit—Department of Biomedical, Dental Science and Morphological and Functional Images, University of Messina, 98100 Messina, Italy; silvana.parisi@unime.it (S.P.); stpergolizzi@unime.it (S.P.); 6 Department of Plastic Surgery, Istituto Oncologico del Mediterraneo, 95029 Viagrande, Italy; corrado.fichera@grupposamed.com; 7 Trauma and Gamma-Knife Center, Department of Neurosurgery, Cannizzaro Hospital, 95126 Catania, Italy; umana.nch@gmail.com

**Keywords:** cutaneous squamous cell carcinoma, radiotherapy, locally advanced cutaneous squamous cell carcinoma, metastatic cutaneous squamous cell carcinoma, inoperable cutaneous squamous cell carcinoma, immunotherapy, chemotherapy

## Abstract

**Simple Summary:**

Nodal and distant metastases of cutaneous squamous cell carcinomas are very rare and lead to dismal prognoses. Immunotherapy is approved only for cutaneous squamous cell carcinoma patients not amenable to surgery or curative radiation therapy. Radiation therapy has a clear role as an adjuvant treatment for locally advanced disease. Radiation therapy may also have an important role in inoperable and metastatic disease. Oligometastatic disease is a condition that needs to be defined for this carcinoma. This review aims to offer to the readers a comprehensive overview of studies about the role of radiotherapy in the management of advanced or metastatic cutaneous squamous cell carcinomas, also assuming possible further developments in the light of the recent discoveries about tumor biology. The present paper has the merit of re-focusing great attention on the efficacy and cost-effectiveness of radiotherapy in these not yet properly explored scenarios.

**Abstract:**

Radiation therapy (RT) is an effective therapeutic option for small localized cutaneous squamous cell carcinoma (cSCC) among patients who are not eligible for or refuse surgery. RT also has a defined role as an adjuvant treatment in cases of adverse features that predispose to tumor recurrence after local excision. Since the development of cSCC is often a late consequence of chronic sun exposure, its occurrence is more common among elderly patients whose comorbidities may contraindicate surgical procedures. These could be impeded not only by frail medical conditions but also by technical issues. Indeed, an aggressive locoregional behavior of cSCC may culminate in unresectability due to widespread invasion of neighboring tissues. Moreover, cSCC could develop distant metastases. Both locally advanced and metastatic cSCCs carry a poor prognosis. In these scenarios, recent discoveries of tumor molecular targets are promoting the use of promising systemic therapies, especially immunotherapy, over RT. However, the results from using immunotherapy and, even more so, of chemotherapy are still not optimal. By contrast, advances in radiation delivery equipment can safely treat even large and complex-shaped cSCC targets in challenging body sites. In addition, RT could also have a role in metastatic cSCC settings by enhancing the effectiveness of concomitant immunotherapy. The aim of this review is to summarize and comment on the body of literature about the use of radiotherapy for operable and inoperable locally advanced cSCCs and for metastatic ones in an attempt to define its current and future role.

## 1. Introduction

Cutaneous squamous cell carcinoma (cSCC) is the second most common skin cancer after basal cell carcinoma (BCC) in white skin populations [1]. cSCC is characterized by a more marked propensity to spread both locoregionally and distantly compared to BCC. Large tumors arising from heavily sun-exposed head and neck areas in immunosuppressed patients are more prone to metastasize [2]. However, the overall low rate (0.4%) of the metastatic stage had held back the development of effective treatment strategies for this scenario until some years ago [3]. Indeed, historically, platinum-based chemotherapy was the only viable option, even though the results were poor [4]. Such a chemotherapy regimen was borrowed from that used for the mucosal counterpart (head and neck squamous cell carcinoma, HNSCC) with which cSCC shares some cancer registries [5]. Recently, greater knowledge of the cSCC mutational burden has revolutionized the treatment of metastatic disease by providing potentially successful immunotherapies [6]. By contrast, the radiotherapy role is well-defined in the adjuvant setting for cSCCs with adverse risk features (e.g., perineural invasion and residual disease) or lymph node metastases [7]. In the metastatic stage, the usefulness of radiotherapy is mainly limited to the palliation of life-altering symptoms. The currently available guidelines advocate the use of immunotherapy (IT) alone for metastatic disease or locally advanced cSCCs that are unlikely to be cured with surgery, radiotherapy or combination treatments, thus creating a gray zone where the role of ’non–curative’ radiation doses is still undefined [8]. As this tumor more frequently occurs among elderly and frail patients, with a tendency to locally recur until surgical therapeutic options are exhausted, the curative intent is sometimes discouraged not only by its extent and previous treatments but also by poor patient compliance or the presence of comorbidities that contraindicate aggressive approaches [9]. Since palliative short-course hypofractionated radiotherapy has proven to be somewhat effective in controlling locally advanced HNSCCs not suitable for curative treatment, its absence in current indications for treating unresectable cSCCs not amenable to classic long-course radiotherapy is at least debatable. Indeed, the effectiveness of hypofractionated radiotherapy for advanced HNSCC has been known for several decades [10] and is still used [11]. Hypofractionated radiotherapy is currently approved for the treatment of small cSCC lesions (<2 cm) when surgery is rejected or contraindicated, as the risk of recurrence drastically increases with increasing tumor size and depth [12]. Actually, some ultra-hypofractionated regimens have been successfully employed, even for larger cSCCs among fragile patients [13]. Nowadays, the promising results obtained by immunotherapy for the treatment of advanced cSCCs have meant that radiotherapy at the primary tumor site was precluded in this patient subset. Even if cemiplimab has substantially changed the prognosis of this stage, the overall response rate is not yet optimal (44%) [14]. In addition, its use in immunosuppressed patients causes some serious concerns about tolerability [15]. Therefore, there is a need to elucidate the current role of radiotherapy in the management of patients with advanced/metastatic cSCCs.

The aim of this review is to investigate the use of radiotherapy in locally advanced or metastatic cSCCs, with an insight to potential future opportunities for integration with immunotherapy.

## 2. Methods

**Figure 1:**
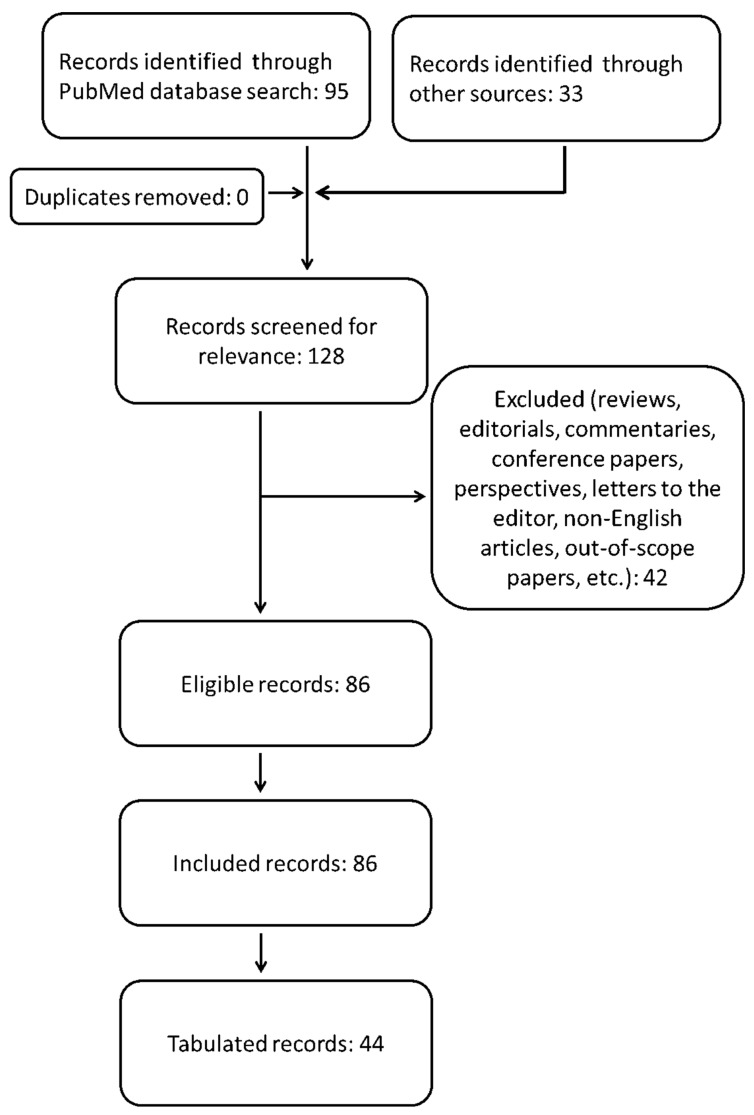
Flowchart of the literature search.

Within the scope of the paper’s topic, we queried the PubMed/MEDLINE database from its inception to 31 December 2021, looking for articles on the use of radiotherapy for the treatment of locally advanced and/or metastatic cutaneous squamous cell carcinoma. We used the following search string: ((“advanced cutaneous squamous cell carcinoma”[Title/Abstract]) OR (“advanced cutaneous squamous cell carcinoma”[MeSH Terms]) OR (“metastatic cutaneous squamous cell carcinoma”[MeSH Terms]) OR (“metastatic cutaneous squamous cell carcinoma”[Title/Abstract])) AND ((“radiotherapy”[Title/Abstract]) OR (“radiotherapy”[MeSH Terms]) OR (“immunotherapy”[Title/Abstract]) OR (“immunotherapy”[MeSH Terms]) OR (“immune checkpoint inhibitors”[MeSH Terms]) OR (“immune checkpoint inhibitors”[Title/Abstract])). Humans and English filters were applied. We also included case reports and small case series due to the rarity of the scenarios investigated here. We discarded duplicates, editorials, comments, reviews and other papers which did not fit the aim of this work. Two authors (G.F. and P.P.) independently reviewed the titles and abstracts of the retrieved literature under the supervision of a third author (S.P.) who resolved any disagreement between them regarding relevance. The references for each paper were checked so as not to overlook any articles appropriate to this investigation. To better orient the reader among the included studies, we arbitrarily chose some of them as deserving of being tabulated for relevance according to our personal perspective (expert opinion). The collection and analysis of bibliographic resources was conducted according to the flowchart shown in Figure 1. All the relevant findings from full-text scanning each paper are summarized in the following paragraphs.

## 3. Operable Locally Advanced CSCC (LACSCC)

### 3.1. The Parotid and Neck Issues

The parotid gland is the main metastatic site for facial cSCC through direct invasion from the overlying skin or, more commonly, the spread of tumor cells to intraparenchymal draining lymph nodes. In a series of 102 parotidectomies for involvement in metastatic cSCC, 86 adjuvant radiotherapy treatments significantly improved local control in the parotid bed with respect to surgery alone. The authors reported only a cumulative 5-year local control rate of 75%, a locoregional control rate of 91% and a disease-specific survival (DSS) rate of 65%. Neither stratification for radiotherapy target extent (neck vs. parotid bed alone) nor information about radiation dose or any systemic therapy were provided. Positive neck nodes worsened survival [16]. By contrast, in another report, the involvement of neck nodes seemed not to have a negative impact on survival among 126 locoregionally advanced cSCC patients of whom 81 had parotid disease: only a greater extent of the latter, single-modality therapy (surgery or radiotherapy) and immunosuppression adversely affected survival. This ranged from 33% to 81%, depending on parotid disease. However, the authors themselves are suspicious of the irrelevance of neck node status. The overall 5-year DSS rate was 68%. Moreover, in this work there were no indications about RT dose and target [17]. Despite the use of combined therapy (surgery plus adjuvant RT) with well-tolerated high radiation doses (60 Gy to the parotid bed and 50 Gy to the ipsilateral neck in 2 Gy/fraction), Dona et al. described a 24% rate of locoregional recurrence, mostly being in-field and within two years of treatment (median 7.5 months), without identifying a predisposing factor (surgical margin status, number of positive neck nodes or extracapsular spread). Experiences such as this gave rise to addressing the issue of the usefulness of any chemotherapy. Interestingly, 16% of clinically negative necks had a positive pathological finding. The 5-year DSS was 72%. Even in this case, no direct comparison was made between radiotherapy to the ipsilateral neck and the parotid bed only for survival and recurrence outcomes [18]. In a work with a small sample size (43 cases of LACSCC originating from the external ear), only 31% of patients treated with combined therapy (surgery plus RT) developed a life-threatening recurrence compared to 38% of those submitted to surgery alone. However, this study was largely too underpowered to draw any definitive conclusions about the significance of the findings [19]. In another study 12/56 patients with a cSCC parotid gland were treated with RT alone, reporting an overall (local and distant) recurrence rate lower than that of the remaining 44 patients treated with combination therapy or surgery alone (17% vs. 27% vs. 57%, respectively) while showing the worst 3-year DSS rate (47% vs. 72% vs. 80%, respectively). This finding confirms that radiotherapy alone is an option only in cases of unacceptable risk for surgery. Facial nerve involvement and a greater extent of parotid disease were significantly associated with a worse prognosis, while neck disease was not. According to the authors, when the first feature is present, radical parotidectomy with facial nerve sacrifice may be required [20].

#### 3.1.1. The Patterns of Relapse despite Radiotherapy

In a study by Southwell et al., adjuvant radiotherapy did not outweigh the greater risk of recurrence and mortality among cSCC patients requiring re-surgery to the parotid gland or neck nodes compared to the previously untreated cases. This entails the careful assessment of cSCC features to maximize the therapeutic effort at first presentation. Again, being immunocompromised and the need for facial nerve sacrifice were poor prognostic factors: the odds ratios (OR) for local and any recurrence were 7.2 and 5.3 with respect to non-immunocompromised patients, while facial nerve involvement increased the risk of local recurrence five-fold [21]. The parotid gland was also involved in 15 out of 27 scalp cSCCs. This form had a poor prognosis, with almost half of all patients developing an early relapse (median 6 months) following regional treatment (surgery and/or radiotherapy) and dying from that (median survival after relapse was 9 months). In regards to the radiotherapy effect on locoregional control, 67% of resection-only patients had regional recurrence as compared to 32% of patients submitted to adjuvant radiotherapy. Among the latter, the regional failure was in-field in 71.4% of cases, despite an RT dose to the operative site equal to 60 Gy in 30 fractions [22]. The use of bolus seems to be unable to improve locoregional control; instead, by determining severe skin reactions to radiation (grade ≥3 radiation dermatitis), its application could lead to long RT interruptions (>6 days) at the expense of effective local control [23]. In a series of 170 patients with locally advanced cSCC, no survival difference between parotid and neck disease alone or concurrently in both sites was detected. The extent of neck node involvement affected survival outcomes only in the absence of parotid disease, but it was not an additional prognostic factor with respect to parotid disease alone. By contrast, a greater extent of the latter significantly worsened survival. In this cohort, where adjuvant RT (range 50–70 Gy) was administered to 77% of patients, the cumulative recurrence rate was 36%: RT was a significant protective factor against cause-specific death (hazard ratio, HR, 0.4), while immunosuppression was a significant adverse factor for survival (HR 3.8) [24].

#### 3.1.2. Facial Nerve Involvement, Positive Surgical Margins and the Debate about the Classification of the Parotid Gland as a Cervical Lymph Node Level

Adjuvant RT might also be useful in preserving facial nerve function in cases of microscopic perineural residual disease. In this setting, Iyer et al. did not report different disease-free survival (DFS) and local recurrence rates with respect to the same treatment protocol for close or negative margins: only 3 out of 15 irradiated patients with microscopic nerve involvement developed a parotid recurrence, and all were amenable to salvage by radical parotidectomy [25]. Such a finding was not confirmed in a much larger study (250 locally advanced cSCC patients), where involved margins, both independently and together with immunosuppression, no adjuvant RT and extra-nodal spread implied a significantly greater risk of death in comparison with clear margins (HR 1.85). In this study, RT improved both locoregional recurrence rates (17% vs. 48%) and survival compared to surgery alone (HR 0.32): the second result could be considered as reflecting the first since most patients with regional recurrence died of disease. Interestingly, in a univariate analysis, the involvement of the parotid gland yielded a worse survival rate compared to neck-only disease, irrespective of the concomitant neck node status. However, this was not confirmed by a multivariate analysis [26]. In opposition to this, Forest et al. proposed and validated across a series of 215 cSCC patients a new staging system in which the parotid gland should be considered as a cervical lymph node level. Indeed, these authors only found that the number of involved lymph nodes from parotid and neck (single or multiple) and their size (< or >3 cm) were predictors of locoregional control and survival. However, even extracapsular nodal spread and RT administration showed a clear trend towards significance as adverse and protective factors, respectively [27].

#### 3.1.3. The Problem of the Occult Disease and How to Face It

The crucial role of parotid gland status in defining the prognosis of locally advanced cSCC is also highlighted by the fact that a fair amount (14.7%) of patients with parotid disease may have occult metastases to the neck (clinically negative nodes); this warrants radiotherapy to the clinically uninvolved ipsilateral cervical lymph nodes [28]. In a recent meta-analysis of 874 patients with parotid metastatic cSCC submitted to elective neck dissection (END), an overall prevalence of occult disease of 22.5% was found, calling for active treatment of the neck in cases of parotid disease. In this review, elective RT alone delivered to the cN0 neck achieved the same oncological outcomes as END without adding any further survival advantage when administered postoperatively but likely avoiding some surgical complications [29]. Radiotherapy associated with extensive surgery (such as lateral temporal bone or facial nerve resections) could mitigate the poorer prognosis of more locally advanced disease by improving local control and ultimately DSS, especially among immunocompromised patients [30]. In another series of 78 patients, adjuvant RT improved 5-year survival and 2-year regional control compared to surgery alone (50% vs. 20% and 89% vs. 40%, respectively). A comparison between elective neck irradiation (ENI), post-END RT and post-therapeutic neck dissection RT was not carried out. In this report, RT significantly lowered the parotid bed recurrence rate (3,7% vs. 27%) so much that a systematic total parotidectomy is highly questionable [31].

#### 3.1.4. The Therapeutic Gain by Radiotherapy over Surgery Alone, Especially in the Presence of Adverse Prognostic Factors

In a large sample of 349 patients with locally advanced cSCC, adjuvant RT improved DSS, especially for those tumors with perineural invasion and regional spread [7]. In another sample of 122 LACSCC patients, adjuvant RT (up to 60 Gy in 30 daily fractions to 102 patients) significantly improved survival outcomes as compared to surgery alone (20 patients). The recurrence rate was 55% for the surgery and 23% for the surgery plus radiotherapy cohorts. The 5-year DFS and overall survival (OS) rates for the two treatment groups were 34% vs. 74% and 27% vs. 66% and were significantly better for the combined treatment compared to surgery alone [32]. The beneficial effect of RT on survival was also highlighted by an even larger sample of regionally metastatic cSCC patients (3534) from a systematic review where, in addition to adjuvant radiotherapy, other prognostic factors were identified: immunosuppression, extracapsular spread, lymph node ratio and advanced age [33]. This latter prognostic factor was not confirmed in another study sample of 442 patients (418 immunocompetent and 24 immunocompromised), with immunosuppression being the predominant adverse factor among frail patients [34]. This promotes the use of aggressive RT, even among elderly patients, just like in other cancers [35,36].

#### 3.1.5. Radiotherapy in the Management of a Limited Burden of Regional Disease

Contrary to earlier reports, a recent study of 101 locally advanced cSCC patients demonstrated that DFS worsens with the increasing number of neck node metastases in an almost-linear fashion. Similarly, patients with more regional metastases were more prone to developing distant metastases. Given that almost all patients (88.1%) were treated with adjuvant RT, this seemed unable to neutralize the effect of involved neck node number on prognosis. However, such inclusive irradiation could lead to underestimating the true adverse impact of the increasing number of lymph node metastases [37]. The latter was confirmed to be prognostically significant in another study, which identified a cut-off of four involved lymph nodes as a discriminating factor for predicting survival [38]. This study with 91 patients disproved the above results [24] regarding the impact of parotid and neck disease on survival outcomes. Indeed, these were negatively affected by the extent of nodal involvement, with the parotid disease alone being a more favorable prognostic factor relative to either isolated neck disease or synchronous neck and parotid disease. According to the authors, this pattern of limited disease deserves a more proactive approach, including, for example, adjuvant radiotherapy that is more specifically tailored to the parotid condition [38]. Indeed, there is a debate over how best to manage patients with potentially limited disease, such as those with a clinically negative regional disease and a high probability of occult metastasis (>19%), as suggested by Wong et al. These authors proposed a decision tree for stage cN0 cSCC of the head and neck, taking into account three different treatment options: surveillance, END or ENI. Active treatment (ENI or END) should be considered when the risk of occult metastasis exceeds 19%. Between 19% and 30% risk, ENI seems to be better than END, probably because radiotherapy is enough to sterilize any micro-metastases. Over that threshold, END may provide better outcomes by offering nodal status data for more effective adjuvant treatment. In decision analysis, the following primary tumor characteristics may be considered as at risk: perineural or lymphovascular invasion, poor differentiation and lesions with increased thickness or horizontal size [39]. The rate of occult parotid metastases was 24.7% in a study by Kadakia and colleagues [40]. However, since Kampel et al. reported the lack of a survival advantage from elective parotidectomy over non-parotidectomy among LACSCC patients with clinically uninvolved parotid glands and positive or negative neck nodes [41], parotid-directed RT could play a role in this patient population by limiting the use of aggressive and potentially dysfunctioning surgery on the parotid gland.

#### 3.1.6. The Effect of Adding Systemic Therapy to Radiotherapy

Moreover, the most recent reports on LACSCC patients claim a survival advantage of adjuvant radiotherapy with an uncertain or nebulous impact of any concomitant chemotherapy and confirm the negative prognostic role of immunosuppression, poorly differentiated histology, primary tumor size >2 cm, extracapsular extension, perineural and/or lymphovascular invasion and positive surgical margins [42,43,44,45]. A single-arm, prospective, phase-1 open-label study tested a combination of erlotinib with fractionated radiotherapy (60–66 Gy in 30–33 fractions), given that the epidermal growth factor receptor (EGFR) may be overexpressed in 56–58% of cSCCs [34,46]. Such a trial involved the administration of erlotinib (150 mg/d) along with 6-week post-operative RT in 15 LACSCC patients after maximal tumor resection. At the cost of a high toxicity rate (73% grade 2–3 acneiform-type rash, 87% grade 2–3 mucositis and other grade 2–3 toxicities, such as esophagitis (40%), fatigue (47%), nausea/vomiting (47%), dehydration (47%) and diarrhea (20%)) requiring dose adjustments or treatment discontinuation in almost half of patients (46.7%), concomitant erlotinib provided no advantage in terms of OS as compared to historical controls treated with radiotherapy alone [47]. This is why the use of erlotinib is not supported by the currently available guidelines for treating this disease stage.

### 3.2. Radiotherapy for Non-Head and Neck cSCCs

Adjuvant RT could decrease the risk of nodal relapse, even among patients with cSCCs arising on the trunk and extremities and progressing to the axilla or groin and should be considered in these rare cases [48,49]. In a sample of 74 patients with axillary metastases, of whom 48 were treated with surgery plus radiotherapy and 15 were treated with surgery alone, the combined treatment achieved the same survival results as surgery alone, but given the significantly more adverse histopathological features and larger lymph nodes (>6 cm), which in themselves carry a greater risk of recurrence and, even ultimately, of death, the remaining 11 patients treated with definitive or palliative radiotherapy had poorer outcomes than those who underwent surgery [50].

All the most significant studies discussed here are summarized in Table 1.

#### Comments

Most studies agree on the usefulness of adjuvant RT in LACSCC patients, and its pivotal role has been supported for at least two decades [51]. Conversely, it is likely that selection bias due to the imbalance of risk factors between patient groups undermines the results of those negative reports that are unable to detect a protective role for radiotherapy.

Whatever the real prognostic value is of parotid and neck involvement, there is some need to define if any adjustments in radiotherapy practice are necessary, such as total dose, dose fractionation or different doses depending on the risk of recurrence at each site (dose escalation to the parotid bed or to any residual microscopic disease or site of extracapsular nodal spread?) and timing with other therapies.

Despite the NCCN guidelines proposing “observation” as a viable option for pN1 cSCC, we recommend some caution regarding this [52]. Such a statement derives from the misleading practice of considering cutaneous forms of SCC as the mucosal counterpart and from experiences such as those of O’Brien et al., where the N2 cSCC patient group had a significantly worse outcome than the N1. However, it should be noted that almost all patients (86%) in this study had postoperative radiotherapy, and therefore no comparison between the pN1 irradiated and non-irradiated patients can be made [53]. Furthermore, in the study of Wang et al., pN ≥ 2 was prognostically worse than pN1 when taking into account the entire cohort (102 patients treated with surgery plus RT and 20 submitted to surgery alone). Additionally, the authors mentioned that none of the four low-risk pN1 cases in the surgery-alone group (20 patients with a 55% recurrence rate) failed locoregionally or distantly. However, such a finding is too small to be significant, asserting that low-risk pN1 LACSCC patients surely do not benefit from adjuvant RT [32]. In the absence of prospective trials clarifying this issue, we believe that regional adjuvant radiotherapy is to be strongly considered for any patient with a pathological finding of neck disease, irrespective of its extent (N1 vs. N2), especially in cases of other adverse features. The presence of the latter may also inform a decision about the need for ENI in undissected cN0 patients.

Among the high burden of cSCC gene alterations [54], there is also over-expression of PD-L1, which, by binding its receptor (PD-1) onto tumor-infiltrating lymphocytes, enacts an immune escape. Since PD-L1 expression was found to be further increased on other tumor cells (basal cell, breast and ovarian carcinomas) as a direct result of irradiation itself, it can be assumed that its blockade by a specific antibody could enhance RT efficacy, even in LACSCC patients [55]. Moreover, PD-L1 expression was found to inversely correlate with tumor differentiation and a tendency to disease progression in the form of regional recurrences or distant metastases. The higher PD-L1 expression of poorly differentiated cSCCs, especially if >90%, seems to be associated with a greater intratumoral and peritumoral cytotoxic CD8+ T-cell density, thus reflecting a stronger host immune response against tumors and predicting a better clinical outcome following local therapies, such as surgery and RT. This could help to personalize treatment on the basis of such clinicopathological features, for example, by tailoring an escalated radiation dose on biomarker-based survival predictions or combining RT with specific immune checkpoint inhibitors (ICI) [56].

Adjuvant RT should also be integrated in the treatment paradigm for LACSCC arising in the non-head and neck regions.

## 4. Inoperable LACSCC: Definitive Radiotherapy Combined or Not with Systemic Therapies

### 4.1. The Combined Treatments Borrowed from the HNSCC Management

Platinum-based chemotherapy (Pt) and cetuximab (Cx) are two systemic therapies that are often used in association with radiotherapy for treating patients with locally advanced mucosal SCC of the head and neck region. This has paved the way for testing the above drugs, even in the cutaneous form of SCC, combined with either adjuvant or definitive radiotherapy to increase its efficacy. Such treatment strategies were adopted in a sample of 23 LACSCC patients, 11 of whom were deemed inoperable and treated with definitive radiotherapy (70.29 Gy in 33 daily fractions) associated with Pt or Cx. Overall, survival outcomes were poor, with 16/23 patients experiencing locoregional recurrence or distant disease progression at a median follow-up of 24 months. Ten deaths were registered as a consequence of cancer progression. No comparison was made between the adjuvant and definitive setting but only between the Cx-group and Pt-group, which achieved the same results in terms of 2-year disease-free and of overall survival (50% vs. 30% (*p* = 0.25) and 73% vs. 40% (*p* = 0.32), respectively). The lack of a control arm treated with radiotherapy alone and the need for modifications and/or deleterious delays in systemic therapy or radiation treatment due to certain clinical problems in more than half of patients (13/23), as Tanvetyanon et al. already previously feared [57], did not support the routine use of these treatment protocols in inoperable LACSCC cases [58]. Actually, RT + Cx gave an initially enthusiastic disease control rate of 91% (complete response (CR) 36%, partial response (PR) 27% and stable disease (SD) 28%), which did not last long, having reported a median PFS of 6.4 months, resulting in a median OS of 8 months in a sample of 12 elderly patients, most of whom had moderate or severe comorbidities and/or immune dysfunction (75%). At a median follow-up of 7 months, median DSS was not reached while 2-year DSS and OS were 51% and 40%, thus suggesting short-term effectiveness of the treatment and a negative impact of poor patient baseline condition. To further complicate these results, there was an 83% grade ≥3 adverse event rate, requiring hospital admission in 67% of cases [59]. Joseph et al. encouraged large trials with RT + Cx based on the results of their small prospective sample: out of eight elderly and frail inoperable LACSCC patients submitted to a curative-intent combined treatment, at a median follow-up of 25 months six had a lasting CR (one of whom died for a treatment-unrelated cause 31 months after therapy) and two had disease progression treated with palliative chemotherapy. The 2-year progression-free (PFS) and cause-specific survival were 83.3% and 87.5%. The treatment was well-tolerated, with acute acneiform rash (characteristic of EGFR inhibition) being the only grade ≥ 3 toxicity in four patients [60]. A 2-year OS rate of 58% was registered by Nottage et al., who treated 21 inoperable LACSCC patients with definitive RT (70 Gy in 2 Gy/fraction to primary tumor site and nodal gross disease and 50 Gy in 2 Gy/fraction for prophylactic nodal irradiation of the cN0 neck) and concomitant weekly cis- or carboplatin. Interestingly, these authors reported a 52.6% CR rate, which lasted in almost all cases, thus positively affecting their survival with respect to those with only partial responses (47.4%). However, even in this prospective trial, there is no radiotherapy-alone arm. While the planned RT was quite safely administered to all patients, chemotherapy was required to be modified or definitively suspended in 12/21 patients, 1 of whom had serious nephrotoxicity requiring permanent dialysis [61].

### 4.2. Immunotherapy or Chemotherapy Drugs to Be Associated with Radiotherapy

The above finding renews concerns about the suitability of chemoradiotherapy (CRT) in this scenario. The data from the above study should be compared with that from the phase 1 trial by Migden et al. that exclusively employed the anti-PD-1 antibody cemiplimab in inoperable LACSCC patients. In Nottage’s work, at 6 months 13/21 (61.9%) patients had locoregional control, including 10 with CR after CRT, 2 disease-free and 1 with a residual tumor after salvage surgery following CRT. None of the 10 patients with CR developed in-field recurrences, as there was only one distant failure and one local relapse outside the RT field. All patients that were incompletely responsive to CRT died of their disease. The first disease response evaluation was 8–12 weeks after the end of treatment (mainly supported by PET imaging). Unfortunately, the duration of response was unspecified. In a partially comparable cohort of 26 patients (18 with locally advanced disease alone and 8 with distant metastases), Migden et al. reported a partial response and stable disease in 50% and 23% of cases resulting in durable disease control (no progression for at least 15 weeks) rate of 65%. The disease response was documented at a median of 2.3 months and lasted at least 6 months in 54% of responsive patients. Nevertheless, it is worth noting that the rate of immunotherapy discontinuation was 53.8% because of disease progression (seven cases), adverse events (two), patient or investigator decisions (three), or death (two, likely unrelated to treatment). Grade ≥3 treatment-related adverse events were registered in 19.2% of patients. Most of the patients (20/26) had previously received radiotherapy for cSCC, which limited the possibility of further irradiation [6]. In a heterogeneous cohort of 195 stage III and stage IV cSCC patients, Amaral et al. described a group of 50 inoperable cases that were treated with RT alone (18), systemic therapies (20) or best supportive care (BSC) (12). These authors reported only OS for the entire cohort while omitting any information about cause-specific survival due to their assumption that most patients, usually elderly and with multiple comorbidities, die of cancer-unrelated causes. Given this premise, they found a favorable trend for systemic treatment compared to RT in the inoperable group (*p* = 0.083). Such a finding is inconclusive without data about the toxicity of each treatment. Interestingly, among operated patients, candidates for further non-systemic treatment for disease recurrence or progression, the ones submitted to RT alone had a worse survival than those treated with re-surgery ± RT, as expected, but also with respect to patients treated with BSC [62]. This highlights the mainly palliative potential of RT when administered alone. Furthermore, chemotherapy and EGFR inhibitors alone also had very poor results, as much in metastatic cSCC as in inoperable LACSCC patients in a study by Cowey et al. who reported a 2-year OS of about 30% in both groups [63]. The curative value of RT may be enhanced by the concomitant administration of chemotherapy as a radiosensitizer. Indeed, in a retrospectively collected sample of 130 patients, including 16 with only locally extended cSCC, 70 with regional lymph node spread and 44 with distant metastases that were treated with platinum-based or non-platinum-based chemotherapy associated or not with concomitant RT, Ogata et al. showed significantly better results for combined therapy than systemic therapy alone. In this comparison, the median PFS, 5-year PFS rate, median OS and 5-year OS rate were 8 months vs. 3 months, 29% vs. 8%, 23 months vs. 12.1 months and 42% vs. 15%, respectively. Moreover, in a subgroup analysis, chemoradiotherapy improved survival outcomes only among patients with local or regional disease, reaching significance in the latter case (median OS 110 months vs. 14 months and 5-year OS rate 54% vs. 21%, respectively). No survival difference was documented in the distantly metastatic setting (median OS 11 months vs. 8.8 months and 5-year OS rate 11% vs. 4%, respectively). This study failed to identify the most effective and safe chemotherapy regimen to associate with RT [64].

### 4.3. The Research Efforts to Maximize the Efficacy of Radiotherapy by Using New Radiosensitizers or Fully Combining with Chemotherapy and Immunotherapy

Other than chemotherapy drugs to increase tumor responsiveness to radiation, new molecules could be of some use. For example, thulium oxide nanoparticles have proven to be effective radiosensitizers in vitro. Their ability seemed not to be enhanced by the addition of platinum compounds. This means that if their being inert in normal healthy tissues and their selective uptake into tumor tissues are confirmed in vivo, encouraging results from their clinical use may be expected while limiting the need for potentially toxic and difficult-to-manage chemotherapies [65]. A new research field in the treatment of inoperable LACSCC is the triple combination of RT, chemotherapy and immunotherapy, which is investigated in the ongoing single arm phase II CRIO trial. This involves the accrual of 15 patients treated with durvalumab (anti-PD-L1 checkpoint inhibitor) administered concurrently and adjuvantly to concomitant platinum-based chemoradiotherapy (70 Gy in 35 daily fractions). The recruitment is expected to end in February 2022 and the study endpoints are about the safety and efficacy of the treatment [66].

### 4.4. The Feasibility of Hypofractionation of Radiation Dose

Lastly, hypofractionated schedules could be equally effective and more attractive than long-course RT for elderly patients with poor compliance to treatment prescription. In particular, we found four different approaches on this issue in the literature. Lavaud et al. described two impressive and long-lasting CRs (>14–16 months) in a sample of four patients with inoperable LACSCC of the head and neck regions treated with pembrolizumab and concomitant hypofractionated RT (26 Gy in four fractions). One of the two experienced a complete regression, even at the level of bone and leptomeninges involved by the disease at presentation. By contrast, two patients had a rapid progression. The median DFS and OS were 14.4 and 15.6 months. There was no toxicity. The authors attributed the two CRs to the immunogenic function of RT favorably interacting with IT [67]. De Felice et al. treated 18 elderly patients with definitive weekly hypofractionated RT (8 Gy once a week per 7–8 weeks), reporting an overall response rate at 12 weeks of 95.7% with excellent symptom relief. The 1-year PFS and OS were 58.7% and 66%. Again, hypofractionation of the radiation dose was well-tolerated [68]. The QUAD shot is a treatment protocol that is mainly used for incurable HNSCC, either as first-line or last-line treatment in previously irradiated patients. However, some reports also include advanced skin cancers. This RT scheme involves the administration of 3.7 Gy twice a day with intervals of at least 6 h for 2 consecutive days (14.8 Gy in four fractions) to be repeated every 3–4 weeks, for a total of four cycles with no concurrent systemic therapy. This treatment can achieve a tumor response rate of up to 85%, with minimal toxicity in radiation-naïve patients (at most grade 2 acute toxicities) and acceptable in re-irradiated ones (10.8% of grade 3 toxicities) [69,70]. Finally, among a large sample of 106 frail and/or elderly patients (median age 86 years) with unresectable or medically inoperable head and neck skin cancer, also including BCC, Merkel cell carcinoma and melanoma treated with high-dose stereotactic body radiotherapy (SBRT) (most commonly 40 or 45 Gy in five fractions twice a week), Voruganti et al. described a cohort of 62 radiation-naïve cSCCs. In the latter case, the 1-year and 2-year OS rates were 44% and 26%, while the 1-year and 2-year PFS rates were 60% and 44%. Considering the whole cohort, the above dose prescriptions achieved an objective response rate of 79% (46% CR and 33% PR). In total, 33/106 patients developed acute grade 3 or 4 treatment-related toxicity, which was unrelated to dose size (≤40 Gy vs. >40 Gy). By contrast, the highest biologically effective doses (BED_10_) were significantly associated with an increased risk of late grade ≥3 toxicity (the overall 1-year rate was 7%), calling for greater caution when adopting these extremely hypofractionated schedules [71]. No trial directly compared hypofractionated radiotherapy with the classic one.

### 4.5. Brachytherapy

Medically inoperable CSCCs or those requiring mutilating surgery with serious impairment of cosmesis and related quality of life may also benefit from the use of brachytherapy. This conservative treatment could be particularly useful in challenging face areas, where the radiation dose scattered by external beam radiotherapy raises concerns about the tolerance of very nearby OARs (i.e., lens, eye and brain), even surpassing the high-accuracy features of stereotactic radiotherapy [72]. In these contexts, brachytherapy allows a very steep dose falloff around the target, drastically limiting the radiation exposure of the neighboring OARs [73]. For example, Tagliaferri et al. reported promising results in terms of 5-year local control, ranging from 69% to 97%, and cosmesis (good in about 80% of cases) in a pooled cohort of patients affected by cSCC of the nasal vestibule [74]. These results are concordant with those recently reported by Taylor et al. in another series of 19 patients with facial skin cancers [75].

Brachytherapy is a valuable approach, even for the treatment of non-facial cSCCs, as demonstrated by Kim et al. who successfully cured a case of cSCC of the hand. In this case, a combined interstitial and surface high-dose-rate brachytherapy treatment preserved hand function and avoided a demolitive surgery but with acute and late sequelae to be carefully pondered [76]. Alpha-emitter brachytherapy might also trigger the abscopal effect. By using this technique, Bellia et al. reported a complete response in both the seeded cSCC of the lower limb and in two further untreated synchronous skin lesions [77].

Brachytherapy is a feasible therapeutic option for cN0 cSCCs in cases of contraindications to surgery for poor clinical conditions or cosmetic issues. However, the specific skills required and the limited availability of this peculiar type of radiotherapy in RT departments may explain the greater use of EBRT.

All the most significant studies discussed here are summarized in Table 2.

#### Comments

Medically or technically inoperable LACSCC has a poor prognosis. Its treatment revolves around curative RT. This generally has limited impact when administered alone, as it is unable to prevent local recurrence and distant disease progression. Currently, there is no level 1 evidence supporting the combination of RT with systemic therapy as a radiosensitizer. Almost all the studies on this issue are retrospective, small-sized and burdened by selection biases and high heterogeneity. The most tested drugs in association with RT are platinum-based compounds and EGFR inhibitors. Neither proved to be better than the other. In addition, both combinations may provide temporary responses. Moreover, their use could result in serious adverse events affecting patient compliance or even survival. However, in the absence of more effective alternatives, these treatment approaches could be considered in clinical practice. This background led to experimentation with new drugs, especially immune checkpoint inhibitors, to obtain better results in terms of survival outcomes and treatment safety. In 2018, the FDA approved the use of cemiplimab for advanced cSCCs that are ineligible for curative treatments. This should postpone its use until after RT failure. There is some need for prospective trials clarifying if and what chemotherapy and EGFR-inhibitor regimens are appropriate and, for others, investigating combinations with new drugs, especially with those oriented at eliciting immune responses against tumors.

Last but not least, it should be pointed out that the concepts of “unresectable” and “untreatable by curative RT” may relate to an inadequate familiarity with the complex anatomy of some skull tumor sites and with the specific expertise required to safely approach them. Indeed, the marked tendency to perineural spread along the cranial nerves [78] sometimes calls into play the intervention of neurosurgeons with highly qualified skills [79]. Furthermore, cSCCs arising on the scalp may have full-thickness invasion of the calvarium, which requires craniectomy followed by cranioplasty [80]. Moreover, in this disease scenario, adjuvant RT seems to confer a survival advantage, as indicated by Kadakia et al. who reported a 3-year DFS and OS of 80% and 62% for irradiated patients (45) and of 62.5% and 32.5%, respectively, for patients submitted to surgery alone (8) [81]. Recent technological advances have been able to assist the neurosurgeon in planning cranial reconstruction as well as the availability of increasingly better performing radiotherapy equipment to allow the radiation oncologist to deliver high precision RT treatment to tumor recurrences around named cranial nerves [82,83]. Target size, site and shape are three different factors that can make radiation treatment difficult. However, new advances in RT techniques can satisfactorily treat even extremely complex skin targets [84,85,86]. Awareness of such therapeutic possibilities may call into question the “incurability” of a large proportion of LACSCCs that could still be amenable to curative-intent treatment. Indeed, there is an urgent need to improve the selection of patients with locally advanced CSCC who are likely to benefit from locoregional treatments and/or systemic cancer immunotherapy [87]. In addition, it is worth pointing out that complex cases have to be managed in high-volume or academic centers because better clinical results in patients treated by specialists with expertise have been demonstrated [88]. A multidisciplinary discussion, including neurosurgeons, otolaryngologists, plastic reconstructive surgeons, radiologists and medical and radiation oncologists is necessary in these complex cases.

## 5. Distantly Metastatic CSCC (M1 CSCC): Does Radiotherapy Have a Role?

The PD-1/PD-L1 axis is effectively targeted by some specific antibodies (ICI), with the most commonly used in cSCC being cemiplimab. Other PD-1 blockade agents approved for unresectable or metastatic cSCC are pembrolizumab and nivolumab. However, cemiplimab seems to be more effective than platinum-based chemotherapy, EGFR inhibitors and pembrolizumab [89,90,91,92]. Objective responses are inconstant and sometimes temporary, with a high propensity for progression: the objective response rate was 31.5%, with a progression rate of 59% and a 1-year OS rate of 46.1% in a sample of 61 patients, irrespective of age and immune status [93]. These results were contradicted by those reported in another slightly smaller sample of advanced cSCC patients (46). In this case, the authors described an overall response (CR+PR) rate of 58.7% (rising to 80.4% when including the ‘stable disease’ outcome), disease progression in 41% of cases and impressive actuarial 1- and 2-year OSs of 79.3%% and 67.1%. Interestingly, non-responders were more common among the distantly metastatic subgroup in the first (61) than in the second samples of patients (46). Grade ≥3 immune-related adverse event rates were 20% and 13%. The second study demonstrated a significantly worse response to immunotherapy for non-head and neck primary tumor locations: almost all advanced cSCCs arising on the trunk or extremities had a rapid progression, culminating in a very limited OS (median 3.8 months) [94]. Such a finding was consistent with that reported by In et al. in a sample of 26 patients with objective response and stable disease rates of 42.3% and 23.1%, which were negatively affected by non-head and neck primary tumors [95]. An alarming grade >3 toxicity rate of 19.2% was reported, which was considerably higher than in other larger studies [96]. Based on the results of the study by Tam et al., RT could also have a curative role in cSCC patients with distant metastases. After all, locoregional RT, variously combined with systemic therapy (mostly platinum-based chemotherapy or an EGFR inhibitor), had a clear beneficial effect on survival outcomes not only among 109 M0 patients but also in 20 M1 patients. Indeed, the latter cohort exhibited significantly worse median OS and 2-year DSS compared to M0 patients (13 months vs. 22 months and 38.2% vs. 57.9%, respectively), but on subgroup analysis, the 8/20 patients who received locoregional RT had better median OS and DSS compared to the remaining M1 patients treated with systemic therapy alone (13 months vs. 7 months, *p* = 0.044 for OS and *p* = 0.125 for DSS) [97].

All the most significant studies discussed here are summarized in Table 3.

### Comments

M1 cSCC has a dismal prognosis. Its rare occurrence (<5% of all cSCC) limits the possibility of setting up prospective clinical trials with adequate accrual and appropriate duration times to test new treatment strategies. The research on PD-L1 and its receptor PD-1 have revolutionized the treatment of this disease stage, enabling us to offer somewhat effective therapeutic options to patients with advanced and metastatic cSCC that is ineligible for local curative approaches (surgery and/or radiotherapy). Therefore, cemiplimab is favored over both platinum-based regimens and EGFR inhibitors. However, even in the best series, it does not achieve an objective response rate exceeding 50–60% against a non-negligible risk of life-threatening adverse events, especially in immunocompromised patients. Immunosuppression is a predisposing factor of cSCC and could make cancer management difficult since the administration of ICIs generates serious concerns about patient safety [15]. In fact, immunotherapy may evoke allograft rejection in solid organ transplant recipients [98,99]. Such considerations call for new safer treatments or the intervention of pre-existing therapeutic opportunities, and RT may be appropriate. Its use is mainly palliative in M1 patients, even though some anecdotal experiences report survival benefits [97]. RT could be curative by integrating it with other local and systemic therapies in this patient population [100]. Moreover, oligometastatic status is a disease stage that is supposed, for other cancers as well as for the mucosal counterpart of cSCC [101,102,103,104,105,106], to indicate an intermediate prognosis between locoregional and plurimetastatic disease extent for patients with a limited number of distant metastases (≤5). Although oligometastases have not yet been defined for cSCC, they could be reasonably postulated. This would encourage the use of ablative stereotactic RT (SBRT) for M1 patients with a low tumor burden to delay the need for potentially toxic immunotherapy. Indeed, SBRT generally has a favorable therapeutic index and could be better tolerated than systemic therapies, including IT, so its use is being tested, even for a number of metastases >5 [107,108]. RT is the only viable therapeutic option for extremely challenging disease sites requiring urgent treatment [109]. The characteristic high doses employed in SBRT, also when spatially fractionated, may trigger beneficial abscopal and bystander effects, which could enhance the immune response against tumor cells in association with IT [110,111]. In some cases, activation of the immune response can cause abnormal findings on follow-up imaging [112,113]. To discern these and assess disease response, the PERCIST criteria can provide additional and more reliable information compared to the RECIST ones [114]. Nevertheless, RT could overcome the immune resistance of non-responders [93]. Despite IT having recently been approved only for treating LACSCC in which surgery or curative RT are not feasible or for treating metastatic disease, a certain number of patients in the studies in the above paragraphs had not received any prior RT and the reason why is omitted. We are aware of some clinical conditions in which RT is risky, unfeasible or even contraindicated, such as re-irradiation, poor patient compliance (especially in cases of long-course RT schedules) and uncontrolled connective tissue or skin diseases [115]. Considering that the feasibility of limited re-irradiation is already well-known for HNSCC, effective short hypofractionated schedules and general indications for the safe management of elderly and frail cancer patients are available [111,116], there is a strong possibility that RT may be judiciously proposed to an increasingly larger patient population. For these reasons and the less-than-optimal results achievable with immunotherapy, it would be preferable not to use ICI upfront but only following RT failure or absolute contraindication. In this era of immunotherapy research for cancer treatment, new and clear evidence supporting a not merely palliative role for RT in M1 cSCC patients should be investigated in ad-hoc designed prospective trials. 

It is important to underline that, in cSCC, the environmental contribution of tumor-associated non-neoplastic cells is being increasingly recognized. Some of the mechanisms that confer pro-tumorigenic properties to cancer-associated fibroblasts (CAFs) have been recently elucidated [117,118]. For example, it has been observed that the modulation of transforming growth factor beta-associated signaling regulates the invasive properties and epithelial-mesenchymal transition in cSCC cells, while fibroblast growth factor influences macrophage infiltration in human dermal fibroblasts. The pharmacological modulation of these molecular routes may represent interesting candidate therapeutic approaches. While some of the signaling pathways associated with the activation of fibroblasts have been described, the impact of RT on these phenomena is yet to be elucidated. However, according to the biology of these signaling mechanisms, the possible use of combined therapeutic strategies may open exciting new scenarios in which the inhibition of CAF activation can synergistically promote the therapeutic effects of RT and ease cancer-induced morbidity.

## 6. Limitations

Almost all studies are retrospective and then influenced by selection bias. To make matters worse, there is a large heterogeneity in the methods of reporting data about radiotherapy: sometimes delivered doses are not specified as well as target extent, RT timing with respect to any associated systemic therapy and RT-related toxicities. Even oncological outcomes are reported in different ways: not all authors indicate OS, DSS, DFS and PFS, thus affecting the possibility of correctly evaluating and comparing the RT effect on disease-specific outcomes between studies. In some of these, there is no distinction in classifying local (in-field) and locoregional (in-field and out-of-field) recurrences, thus impeding the assessment of the effect of differential doses to varying at-risk RT targets (the primary tumor site, parotid bed and neck nodes). RT techniques and doses have varied from the beginning of the century (50–60 Gy by 3D-CRT) to those currently employed (60–70 Gy by IMRT), which introduces a chronological bias. Most studies have a very small sample size. This is why even similarly designed studies may provide contradictory results. The lack of granular data on patient characteristics and treatment prevents any comprehensive analysis. Many case series, including the few prospective ones [47,60,61,66], are largely underpowered to draw any definitive conclusion and have mainly descriptive/narrative value, just like case reports. All the above limit leading a meta-analysis and advise caution in interpreting the reported results, which currently advocate using RT for advanced cSCCs mostly with the lowest levels of evidence (3–5).

## 7. Conclusions

Radiotherapy has a key role in treating LACSCC, both in adjuvant and definitive settings. The studies discussed here seem to support its beneficial effect on survival outcomes, even among inoperable patients. RT might have a fundamental role, even in M1 cSCC patients, thanks to its immunogenic effect on tumor microenvironments. New integrations with immunotherapy or other systemic therapies are necessary to empower its curative function. The weakness of the currently available literature evidence advocates the need for large prospective clinical trials.

## Figures and Tables

**Table 1:** Overview of studies including patients with operable locally advanced cutaneous squamous cell carcinomas.

	Authors Year	Study Size	Surgery Type No. Patients (Percentage)	Radiation Protocol No. Patients (Percentage)	Systemic Therapy No. Patients (Percentage)	Outcomes	Adverse Events (Grade ≥ 3) No. Patients (Percentage)
1	Bron et al.—2003 [16]	101	Parotidectomy 101 (100%) Neck dissection 75 (74.3%)	EBRT 101 (100%)	N/A	LC 5-year 94% DSS 5-year 65%	N/A
2	Dona et al.—2003 [18]	74	Parotidectomy 74 (100%) Neck dissection 52 (70.3%)	EBRT Parotid 74 (100%), Neck 56 (75.7%)	N/A	LC 2-year 76%; 5-year 73%	0 (0%)
3	Palme et al.—2003 [17]	126	Parotidectomy 88 (69.8%) Neck dissection 87 (69%)	EBRT 126 (100%)	N/A	LC 5-year 80% DSS 5-year 68%	N/A
4	Audet et al.—2004 [20]	56	Parotidectomy 44 (78.6%) Neck dissection 28 (50%)	EBRT 56 (100%)	N/A	DSS 3-year 72% Recurrence 29%	N/A
5	Southwell et al.—2006 [21]	49	Parotidectomy 46 (93.9%) Neck dissection 43 (87.8%)	EBRT 49 (100%)	N/A	Recurrence 56% OS 1-year 88%; 2-year 80%	N/A
6	Ch’ng et al.—2008 [24]	170	Parotidectomy 135 (79.4%) Neck dissection 150 (88.2%)	EBRT 170 (100%) 50–70 Gy	N/A	DFS 5-year 59% DSS 5-year 69% OS 5-year 48% Recurrence 36%	N/A
7	Howle et al.—2008 [22]	27	Parotidectomy 16 (59.3%) Neck dissection 29 (96.3%)	EBRT 27 (100%) 60 Gy in 30 fractions	N/A	Recurrence 48% PFS 6 months (2–29) OS 9 months (1–73)	N/A
8	Iyer et al.—2009 [25]	176	Parotidectomy 176 (100%) Neck dissection 136 (77.3%)	EBRT 176 (100%) 54 Gy (45–66) in 27 fr	N/A	LC 5-year 80% OS 5-year 60%	N/A
9	Oddone et al.—2009 [26]	250	Parotidectomy 152 (61%) Neck dissection 223 (89.2%)	EBRT 250 (100%) 60 Gy (50–74) in 30 fr	N/A	Recurrence 28% PFS 8 months (2–34)	N/A
10	Forest et al.—2010 [27]	215	Parotidectomy 198 (92.1%) Neck dissection 166 (77.2%)	EBRT 215 (100%) 54 Gy parotid, 50 Gy neck	N/A	OS 2-year 82%: 5-year 69% DSS 2-year 87%; 5-year 77% LC 2-year 81%; 5-year 73%	N/A
11	Goh et al.—2010 [48]	26	N/A	EBRT 26 (100%) 50 Gy (45–66)	Chemotherapy 2 (7.7%)	Recurrence 27% PFS 2.2 months (0.5–14.1) OS 18.5 months (0.5–74.5)	N/A
12	Turner et al.—2010 [19]	43	Parotidectomy 36 (83.7%) Neck dissection 35 (81.4%)	EBRT 43 (100%) 60 Gy (36–74) in 30 fr	N/A	Recurrence 35% PFS 5 months (4–20) OS 13 months (4–89)	N/A
13	Kirke et al.—2011 [28]	51	Parotidectomy 51 (100%) Neck dissection 34 (66.7%)	EBRT 51 (100%) 60 Gy in 30 fr	N/A	Recurrence 17.6%	N/A
14	Pramana et al.—2012 [23]	75	Parotidectomy 28 (37%) Neck dissection 47 (63%)	EBRT 75 (100%) 60 Gy (42–70) in 28 fr BED 72 Gy (50–84)	N/A	LC 5-year 67% DSS 5-year 66% OS 5-year 52%	Dermatitis 41 (55%) ORN 4 (5%)
15	Sweeny et al.—2012 [46]	56	N/A	EBRT 56 (100%)	N/A	OS 2-year 64%; 5-year 56%	N/A
16	Wang et al.—2012 [32]	122	Neck dissection 122 (100%)	EBRT 122 (100%) 60 Gy in 30 fr	N/A	Recurrence 28% DFS 5-year 56%	N/A
17	Heath et al.—2013 [47]	15	Neck dissection 15 (100%)	EBRT 15 (100%) 60–66 Gy	Erlotinib 15 (100%)	OS 1-year 83%; 2-year 65% DFS 1-year 73%; 2-year 60% Recurrence 26.7%	Dermatitis 10 (67%)
18	Smith et al.—2016 [34]	442	N/A	EBRT 442 (100%)	N/A	Recurrence 17%	N/A
19	Hirshoren et al.—2018 [31]	78	Parotidectomy 78 (100%) Neck dissection 25 (32.1%)	EBRT 78 (100%)	N/A	LC 5-year 76% OS 5-year 46%	N/A
20	Porceddu et al.—2018 [45]	310	Neck dissection 310 (100%)	EBRT 310 (100%) 60 Gy in 30 fr	Carboplatin 153 (49.4%)	DFS 2-year 83%; 5-year 73% OS 2-year 88%; 5-year 79%	Hearing loss 17 (5.5%) ORN 10 (3.2%) Tinnitus 6 (1.9%) Neuropathy 4 (1.3%) Cataract 1 (0.3%)
21	Sood et al.—2019 [37]	101	Parotidectomy 78 (77.2%) Neck dissection 90 (89.1%)	EBRT 101 (100%)	N/A	Recurrence 24.8%	N/A
22	Trosman et al.—2020 [44]	104	N/A	EBRT 104 (100%)	Carboplatin 38 (37%)	OS 2-year 91%; 5-year 82% DFS 2-year 64%; 5-year 64%	N/A
23	Wilkie et al.—2020 [38]	91	Parotidectomy 71 (78%) Neck dissection 20 (22%)	EBRT 91 (100%)	N/A	Recurrence 36.3% PFS 9 months (3–38) OS 42 months (12–104) OS 5-year 43.8% DFS 5-year (36.2%) DSS 5-year 63.8%	N/A
24	Hazim et al.—2021 [43]	21	N/A	EBRT 21 (100%) Photon 11 (52%) 70 Gy Proton 10 (48%) 70 GyRBE	Cisplatin 10 (48%) Cetuximab 2 (10%) Cemiplimab 1 (5%) Paclitaxel 1 (5%)	Recurrence 40.8% PFS 2-year 44.5% OS 2-year 84.8%	Dermatitis 4 (19%) Thrombocytopenia 8 (38%) Mucositis 2 (9.5%)
25	Kampel et al.—2021 [42]	74	Parotidectomy 48 (65%) Neck dissection 63 (85%)	EBRT 74 (100%)	Chemotherapy 7 (9.5%)	OS 5-year 54.1% DFS 5-year 77%	N/A
26	Yang et al.—2021 [50]	74	N/A	EBRT 74 (100%) 50 Gy in 25 fr	N/A	DFS 2-year 49%; 5-year 49% OS 2-year 68%; 5-year 51%	N/A

Abbreviations: BED, Biologically effective dose; DFS, Disease-free survival DSS, Disease-specific survival; EBRT, External beam radiation therapy; LC, Local control; N/A, Not available; ORN, Osteoradionecrosis; OS, Overall survival; PFS, Progression-free survival.

**Table 2:** Overview of studies including patients with inoperable locally advanced cutaneous squamous cell carcinomas.

	Authors Year	Study Size	Radiation Protocol No. Patients (Percentage)	Systemic Therapy No. Patients (Percentage)	Outcomes	Adverse Events (Grade ≥ 3) No. Patients (Percentage)
1	Samstein et al.—2014 [59]	12	EBRT 12 (100%) 60 Gy (12–80) in 30 fr	Cetuximab 12 (100%)	RR 64%; DC 91% DSS 2-year 51% OS 2-year 40%	Dermatitis 2 (16.7%) Thrombocytopenia 2 (16.7%) Mucositis 1 (8.3%)
2	Lu et al.—2015 [58]	23	EBRT 23 (100%) 60 Gy in 30 fr	N/A	Recurrence 12 (52%) PFS 8 months (1-31)	N/A
3	Tanvetyanon et al.—2015 [57]	61	EBRT 61 (100%) 60–66 Gy in 30 fr	Carboplatin or Cisplatin 61 (100%)	Recurrence 50% PS 23.5 months (7.4–39.5)	Leukopenia 3 (4.9%) Mucositis 3 (4.9%) Neurological 3 (4.9%)
4	Nottage et al.—2017 [61]	21	EBRT 21 (100%) 70 Gy in 35 fr	Cisplatin 21 (100%)	LC 1-year 61.9% OS 1-year 80.2% DFS 1-year 100%	Thrombocytopenia 6 (28.6%) Anemia/Fibrosis 5 (23.8%) Hearing loss 4 (19%) Leukopenia/ORN 2 (9.5%)
5	Joseph et al.—2018 [60]	8	EBRT 8 (100%) 55–66 Gy in 22–30 fr	Cetuximab 8 (100%)	DFS 2-year 87.5% PFS 2-year 83.3% OS 2-year 87.5%	Dermatitis 4 (50%) ACS/fatigue/mucositis 1 (12.5%)
6	Cowey et al.—2019 [63]	82	EBRT 82 (100%)	Carboplatin and Paclitaxel 22 (26.8%) Cetuximab 20 (24.4%) Cisplatin and 5-FU 6 (7.3%) Cisplatin 5 (6.1%) CarboP, PacliT and Cetux 5 (6.1%) CisP, Cetux and 5-FU 3 (2.7%) Other 21 (25.6%)	OS 1-year 56.1%; 2-year 30.2%; 3-year 15.6%	N/A
7	Lavaud et al.—2019 [67]	4	Hypofractionated EBRT 4 (100%) 26 Gy in 4 fr	Pembrolizumab 4 (100%)	PFS 14.4 months OS 15.6 months	0 (0%)
8	Fan et al.—2020 [70]	166	Hypofractionated EBRT 166 (100%) Photon 92 (55%) Proton 74 (45%) 45 Gy in 12 fr	Cetuximab 32 (39%) Chemotherapy 30 (36%) Immunotherapy 11 (13%) Combination 10 (12%)	RR 66% OS 1-year 25.3% PFS 1-year 17.7%	Dysphagia 11 (6.6%) Trismus 5 (3%) Dermatitis 3 (1.8%) Mucositis/ORN/OSM 1 (0.9%)
9	Ogata et al.—2020 [64]	130	EBRT 62 (48%)	Carbo/Cisplatin 74 (57%) Cetuximab 5 (3.8%) Other 51 (39.2%)	PFS 5-year platinum 14%, no 22% OS 5-year platinum 29%, no 26% PFS 5-year non-RT 8%, RT 29% OS 5-year non-RT 15%, RT 42% PFS 5-year RT-plat 20%, RT-no 41% OS 5-year RT-plat 25%, RT-no 48%	Skin ulcer 3 (2.3%) Anemia/Hyponatriemia 2 (1.5%) Duodenal ulcer/Heart failure/Febrile neutropenia/Erythema multiforme 1 (0.8%)
10	De Felice et al.—2021 [68]	18	Ultra-hypofractionated EBRT 18 (100%) 56-64 Gy in 7–8 fr	N/A	OS 1-year 66%; 2-year 26.4% PFS 1-year 58.7%; 2-year 23.5%	0 (0%)
11	Voruganti et al.—2021 [71]	77 (out of 106 various skin cancers)	SBRT 106 (100%)	N/A	OS 1-year 44%; 2-year 26% PFS 1-year 60%; 2-year 44%	Dermatitis 31 (29.2%) Mucositis 1 (1%) Skin ulceration 1 (1%) Fibrosis 7 (6.6%) ORN 1 (1%)

Abbreviations: ACS, Acute coronary syndrome; DC, Disease control; DFS, Disease-free survival; DSS, Disease-specific survival; EBRT, External beam radiation therapy; N/A, Not available; ORN, Osteoradionecrosis; OS, Overall survival; OSM, Osteomyelitis; PFS, Progression-free survival; RR, Response rate.

**Table 3:** Overview of studies including patients with metastatic cutaneous squamous cell carcinomas.

	Authors Year	Study Size	Radiation Protocol No. Patients (Percentage)	Systemic Therapy No. Patients (Percentage)	Outcomes	Adverse Events (Grade ≥ 3) No. Patients (Percentage)
1	Foote et al.—2014 [91]	16	Previous EBRT 14 (87.5%)	Panitumumab 16 (100%)	PFS 8 months OS 11 months OS 2-year 37.5%	Dermatitis 4 (25%) Fatigue 1 (6%)
2	Gold et al.—2018 [92]	39	Previous EBRT 32 (82%)	Erlotinib 39 (100%)	DC 72% PFS 4.7 months (3.5–6.2) OS 13 months 88.4–20.5) OS 1-year 53%; 3-year 19%	Fatigue 4 (10%) Dermatitis 3 (8%)
3	Hanna et al.—2020 [93]	61	Previous EBRT 36 (59%)	Cemiplimab/Nivolumab/Pembrolizumab 61 (100%)	PFS 6-month 50.3% OS 1-year 46.1%	Gastrointestinal 5 (8.2%) Rheumatologic 4 (6.6%) Skin 2 (3.3%) Muscular 1 (1.6%) Neurologic 1 (1.6%)
4	In et al.—2020 [95]	26	Previous EBRT 10 (38.5%)	Cemiplimab 13 (50%) Pembrolizumab 7 (26.9%) Nivolumab 6 (23.1%)	PFS 5.4 months RR 42.3% DR 7.6 months (2.8–28.8)	DKA 2 (7.7%) Cardiomyopathy 1 (3.8%) Coagulopathy 1 (3.8%) Pneumonitis 1 (3.8%)
5	Rischin et al.—2020 [96]	115	Previous EBRT 88 (76.5%)	Cemiplimab 26 (100%)	DC 67.8% DR 1-year 90%) OS 1-year 80.7%	Anemia 7 (6.1%) Fatigue 4 (3.5%) Pneumonitis 3 (2.6%) Dyspnea 2 (1.7%) Rash 1 (0.9%)
6	Salzmann et al.—2020 [94]	46	-	Pembrolizumab 28 (61%) Nivolumab 19 (22%) Cemiplimab 8 (17%)	RR 58.7% DC 80.4% PFS 1-year 58.8%; 2-year 52.3% OS 1-year 79.3%; 2-year 67.1%	Myositis 2 (4.3%) Pneumonitis 2 (4.3%) Arthritis 1 (2.2%) Dermatitis 1 (2.2%) Thyreoiditis 1 (2.2%)
7	Hughes et al.—2021 [90]	105	Previous CT-RT 17 (16.2%)	Pembrolizumab 105 (100%)	RR 35.2% DC 52.4% DR 1-year 77.8% PFS 1-year 36.4% OS 1-year 48,4%	Hepatitis 2 (1.3%) Dermatitis 1 (0.6%) Fatigue 1 (0.6%) Nephritis 1 (0.6%) Pneumonitis 1 (0.6%)

Abbreviations: ACS, Acute coronary syndrome; DC, Disease control; DFS, Disease-free survival; DKA, Diabetic ketoacidosis; DR, Duration response; DSS, Disease-specific survival; EBRT, External beam radiation therapy; N/A, Not available; ORN, Osteoradionecrosis; OS, Overall survival; OSM, Osteomyelitis PFS, Progression-free survival; RR, Response rate.

## Abbreviations

cSCC	cutaneous squamous cell carcinoma
BCC	basal cell carcinoma
RT	radiation therapy
HNSCC	head and neck squamous cell carcinoma
IT	immunotherapy
DSS	disease-specific survival
LACSCC	locally advanced cutaneous squamous cell carcinoma
NCCN	National Comprehensive Cancer Network
DSS	disease-specific survival
DFS	disease-free survival
PFS	progression-free survival
OS	overall survival
CR	complete response
PR	partial response
SD	stable disease
3D-CRT	3-dimensional conformal radiotherapy
IMRT	intensity-modulated radiotherapy
SBRT	stereotactic body radiotherapy
END	elective neck dissection
ENI	elective neck irradiation
EGFR	epidermal growth factor
PD-1	programmed cell death protein 1
PD-L1	programmed death-ligand 1
ICI	immune checkpoint inhibitors
FDA	food and drug administration
BED	biological effective dose
Pt	platinum-based chemotherapy
Cx	cetuximab
CRT	chemoradiotherapy
PET	positron emission tomography
RECIST	response evaluation criteria in solid tumors
PERCIST	positron emission tomography (PET) response criteria in solid tumors
